# Pregnancy and neonatal outcomes following statin exposure in early pregnancy: a nationwide consultation-based cohort study in Japan

**DOI:** 10.1186/s12884-026-08932-6

**Published:** 2026-03-14

**Authors:** Izumi Fujioka, Mikako Goto, Tatsuhiko Anzai, Kunihiko Takahashi, Sachi Koinuma, Atsuko Murashima

**Affiliations:** 1 The Japan Drug Information Institute in Pregnancy, Integrated Center for Women’s Health, National Center for Child Health and Development, Tokyo, Japan; 2 Department of Biostatistics, M&D Data Science Center, Institute of Integrated Research, Institute of Science Tokyo, Tokyo, Japan; 3 Department of Rheumatology and applied Immunology, Saitama Medical University, Saitama, Japan

**Keywords:** Statins, Pregnancy, Congenital anomalies, Perinatal outcomes, Hypercholesterolemia

## Abstract

**Background:**

Statins are generally recommended to be discontinued once pregnancy is recognized; however, unintentional exposure during early pregnancy may occur in women receiving long-term lipid-lowering therapy, and clinical decision-making often requires careful consideration of potential risks and benefits. We aimed to evaluate the risk of congenital anomalies detected at 1 month and perinatal outcomes among infants born to women exposed to statins during early pregnancy (4–13 weeks of gestation) in Japan.

**Methods:**

Using data from the Japan Drug Information Institute in Pregnancy database (2005–2017), we compared pregnant women exposed to statins during early pregnancy (statin-exposed group) and unexposed controls. Neonatal congenital anomalies at 1 month among live births were the primary outcome; secondary outcomes included pregnancy outcomes such as live birth, miscarriage, abortion, and stillbirth. Propensity score matching (1:1) was conducted to estimate the associations between statin exposure and pregnancy outcomes, and inverse probability weighting was performed using stabilized weights as a sensitivity analysis.

**Results:**

Among 968 eligible pregnant women, 65 were in the statin-exposed group and 903 in the control group. After propensity score matching, the prevalence of congenital anomalies did not significantly differ between the statin-exposed (1.6%) and control (1.6%) groups, however, estimates were imprecise (odds ratio: 1.00; 95% confidence interval: 0.06–15.99). Increased risks of preterm birth (risk ratio: 4.26 [2.02–8.99]) and low birth weight (risk ratio: 3.32 [1.73–6.36]) were observed in the IPW analysis but not in the primary PSM analysis.

**Conclusions:**

No increase was observed in congenital anomalies detected at 1 month among live births following early statin exposure, although the estimates were imprecise and the study was underpowered for rare outcomes. Higher risks of preterm birth and low birth weight were observed in weighted analyses but not in the primary propensity score–matched analysis. Further research is needed to clarify perinatal risks associated with statin use in pregnancy. These findings may help inform individualized shared decision-making for women who require statin therapy during pregnancy.

**Supplementary Information:**

The online version contains supplementary material available at 10.1186/s12884-026-08932-6.

## Background

 Familial hypercholesterolemia (FH) is a hereditary condition characterized by high cholesterol levels, early-onset coronary artery disease, and the presence of tendon and skin xanthomas. The incidence of FH is approximately 1 in 300 individuals for heterozygotes and 1 in 1 million individuals for homozygotes [1]. Given the high risk of coronary artery disease in patients with hypercholesterolemia, early diagnosis, rigorous treatment, and family screening are crucial for effective management. Furthermore, with the increasing age of childbearing in recent years, the prevalence of hypercholesterolemia during pregnancy is on the rise, influenced primarily by lifestyle factors and the physiological effects of pregnancy [2–4]. The impact of FH or hypercholesterolemia on pregnancy outcomes such as preterm birth, low birth weight, and congenital anomalies remains debated.

Statins, the first-line treatment for hypercholesterolemia treatment [5], have shown a potential teratogenic risk in animal studies and possible neurodevelopmental effects on offspring [6]. Statins are contraindicated for pregnant women by the European Medicines Agency. The labeling describes that if a patient plans to become pregnant, the physician has to be informed immediately, and statins should be discontinued because of the potential risk to the fetus. In contrast, the U.S. Food and Drug Administration (FDA) requested the removal of the strongest warning against statin use in pregnant patients in July 2020. While discontinuing statins is recommended upon pregnancy confirmation in most patients, classifying statins as contraindicated for all pregnant women may not be entirely appropriate in some cases. Individual cases may warrant a detailed evaluation of risks and benefits based on specific circumstances and medical considerations. This is because, in a small number of pregnant patients at high risk, the potential benefits of statins in preventing severe or life-threatening events may outweigh the risks [7].

According to the Japanese Clinical Practice guidelines, lipid-lowering drugs, including statins, should be discontinued at least 3 months before planning pregnancy, except for bile acid sequestrants [8]. Bile acid sequestrants and low-density lipoprotein (LDL) apheresis are considered safe alternatives to statins during pregnancy; however, these sequestrants typically produce only a modest reduction in LDL cholesterol level, which may not be adequate in certain patients. During LDL apheresis, attention should be paid to symptoms such as nausea, bradycardia, and hypotension due to excessive bradykinin production [9, 10]. Moreover, this therapy may be challenging to manage in small-scale hospitals.

Safety information for drugs during pregnancy and lactation is typically described in the pregnancy and lactation section of package inserts. Numerous medications come with stringent restrictions, such as contraindications for pregnant women, which may limit treatment options for women of childbearing age. For instance, in Japan, statins are categorized as “contraindicated” in the package insert for “women who are pregnant, may be pregnant, and breastfeeding.” Notably, although some studies suggest no increased risk of congenital anomalies in infants exposed to statins in the early pregnancy stages [1, 2, 9, 11], the current evidence remains limited, and further studies are warranted to better understand this issue.

To address this gap in knowledge, we aimed to evaluate the risk of congenital anomalies in infants born to women exposed to statins in the early stages of pregnancy and assess the perinatal outcomes of infants exposed to statins using the Japan Drug Information Institute in Pregnancy (JDIIP) database.

## Methods

### Data source

This study utilized data from the JDIIP database, which was established in 2005 as a project of the Ministry of Health, Labour and Welfare of Japan, located within the National Center for Child Health and Development. The JDIIP compiles epidemiological research from both domestic and international sources on pregnant women and those planning pregnancy, providing safety information during pregnancy through face-to-face counseling. With a network of base hospitals in 62 locations across all 47 prefectures, the JDIIP collects and compiles data on cases of mothers who have undergone counseling, along with details regarding their infants at the age of 1 month [12, 13]. The JDIIP has reported on the effects of drug exposure during pregnancy on infants [14–17] and serves as the only large-scale database in Japan providing insights into the impact of drug exposure on infants during the early stages of pregnancy.

The JDIIP database comprises approximately 12,971 participants from October 2005 to December 2017, of which approximately 5,840 cases involve individuals who were pregnant at the time of registration [12]. Before counseling, a structured questionnaire was used to collect information from participants, including the expected date of delivery, pregnancy history, presence of congenital anomalies in previous offspring, medical history, current diseases under treatment, and details of all medications used since the last menstrual period such as daily dosage, start and end dates, as well as other relevant details. Administrators collected missing information via telephone and validated the data through confirmation by healthcare professionals during counseling sessions. For participants who provided consent for follow-up investigations, postcards were mailed 1 month postpartum to verify pregnancy outcomes, including live birth, miscarriage, abortion, or stillbirth. Additional information was obtained regarding the delivery date, gestational age at delivery, and mode of delivery, as well as neonatal measurements, including birth weight, length, head circumference, chest circumference, and any indications of congenital anomalies as reported by the attending physician. Cases of infants suspected to have congenital anomalies were confirmed by physicians, and with the individual’s consent, pediatricians were contacted for further validation. The response rate among participants who received counseling and provided consent for follow-up was 83.1%, representing the overall follow-up rate of the dataset [12].

### Patient selection

Among women registered with the JDIIP between October 2005 and December 2017, pregnant women who provided consent to participate in the survey, reported their pregnancy outcomes, and were not exposed to any known teratogenic agent [14] were included. Women were excluded if they had twin pregnancies, malignant conditions, exposure to known teratogenic drugs, or insufficient information on pregnancy outcomes and potential confounding factors. Known teratogenic drugs were defined as valproic acid, carbamazepine, phenytoin, phenobarbital, cyclophosphamide, methotrexate, etretinate, mycophenolate mofetil, and warfarin [15, 16].

### Exposure

Women who used statins, including pravastatin, simvastatin, rosuvastatin, pitavastatin, atorvastatin, and fluvastatin, during early pregnancy were classified as the exposed group. Early pregnancy was defined as the period from 4 to 13 weeks of gestation. The start date of the last menstrual period (LMP) was estimated based on the reported delivery date and gestational age at delivery. The exposure period for statin use was then calculated using the estimated LMP. For miscarriages, stillbirths, and elective terminations, gestational age at outcome was used when available; otherwise, it was estimated from self-reported LMP and/or the expected date of delivery (EDD) at enrollment. Gestational age for stillbirths was confirmed in detail.

The control group consisted of women who did not use statins during pregnancy. Women who used statins only in the second or third trimester, but not during early pregnancy, were excluded from the control group.

### Outcomes

The primary outcome was occurrence of neonatal congenital anomalies identified at the 1-month check-up. Secondary outcomes included pregnancy outcomes such as live birth, miscarriage, abortion, and stillbirth. Preterm birth was defined as delivery before 37 weeks of gestation, and low birth weight was defined as a birth weight below 2500 g. Analyses of preterm birth and low birth weight were restricted to live births. These definitions were used in the assessment of secondary outcomes.

Information on congenital anomalies was obtained from maternal reports, which included findings documented by pediatricians during the 1-month check-up. These reports were based on records from the Maternal and Child Health Handbook, a comprehensive record of medical examinations and health-related information for each child in Japan, spanning from pregnancy to 6 years of age [18]. Congenital anomalies were classified as major or minor based on the European Congenital Anomaly Monitoring classification system [19]. Pregnancy outcomes were documented for all pregnancies included in this analysis. Information on congenital anomalies among miscarriages, stillbirths, and elective terminations was incomplete and inconsistently reported; therefore, analyses of congenital anomalies were restricted to live births. Miscarriage, stillbirth, and elective termination were analyzed separately as secondary outcomes.

### Statistical analysis

Baseline characteristics of the study participants are summarized as medians with interquartile ranges (IQRs) for continuous variables and as counts (percentages, %) for categorical variables. A propensity score (PS) was created using logistic regression, with statin exposure in early pregnancy as the dependent variable. Age, body mass index (BMI), registration year, alcohol consumption, and smoking were included as independent variables to account for potential confounding factors. In defining alcohol consumption and smoking during pregnancy, women who reported these behaviors before recognizing pregnancy but discontinued afterward were also considered as having exposure during pregnancy. PS matching (PSM) between the statin-exposed and unexposed groups was conducted using a 1:1 ratio with a caliper width of 0.2 and the greedy pair algorithm without replacement. PSM was used as the primary analysis to estimate the association between statin exposure and pregnancy outcomes. Most women who use statins have underlying hypercholesterolemia and are therefore likely to have concomitant maternal comorbidities such as hypertension and diabetes mellitus. In contrast, the prevalence of these comorbidities is expected to be relatively low in the unexposed group. Under these circumstances, including such maternal comorbidities as covariates in the PS model would result in limited common support between groups and a substantial reduction in the number of comparable matched pairs. Therefore, these comorbidities were not included in the primary PS model in this study.

As a sensitivity analysis, inverse probability weighting (IPW) was performed using stabilized weights. The IPW model included the following covariates: age, year of consultation, alcohol use, smoking, parity, BMI, diabetes, hypertension, psychiatric disorders, cardiac diseases, and folic acid supplementation prior to pregnancy. Participants with missing covariate information were excluded from the primary PSM and IPW analyses. No multiple imputation or missing-indicator approach was applied. A complete-case analysis excluding participants with missing covariate information was performed as a sensitivity analysis and is presented in the Supplementary Material (Table S2). Covariate balance before and after PSM was assessed using standardized mean differences (SMDs) (Table 2). All statistical analyses were performed using R, version 4.1.1 (R Foundation for Statistical Computing; Vienna, Austria).

## Results

**Fig. 1:**
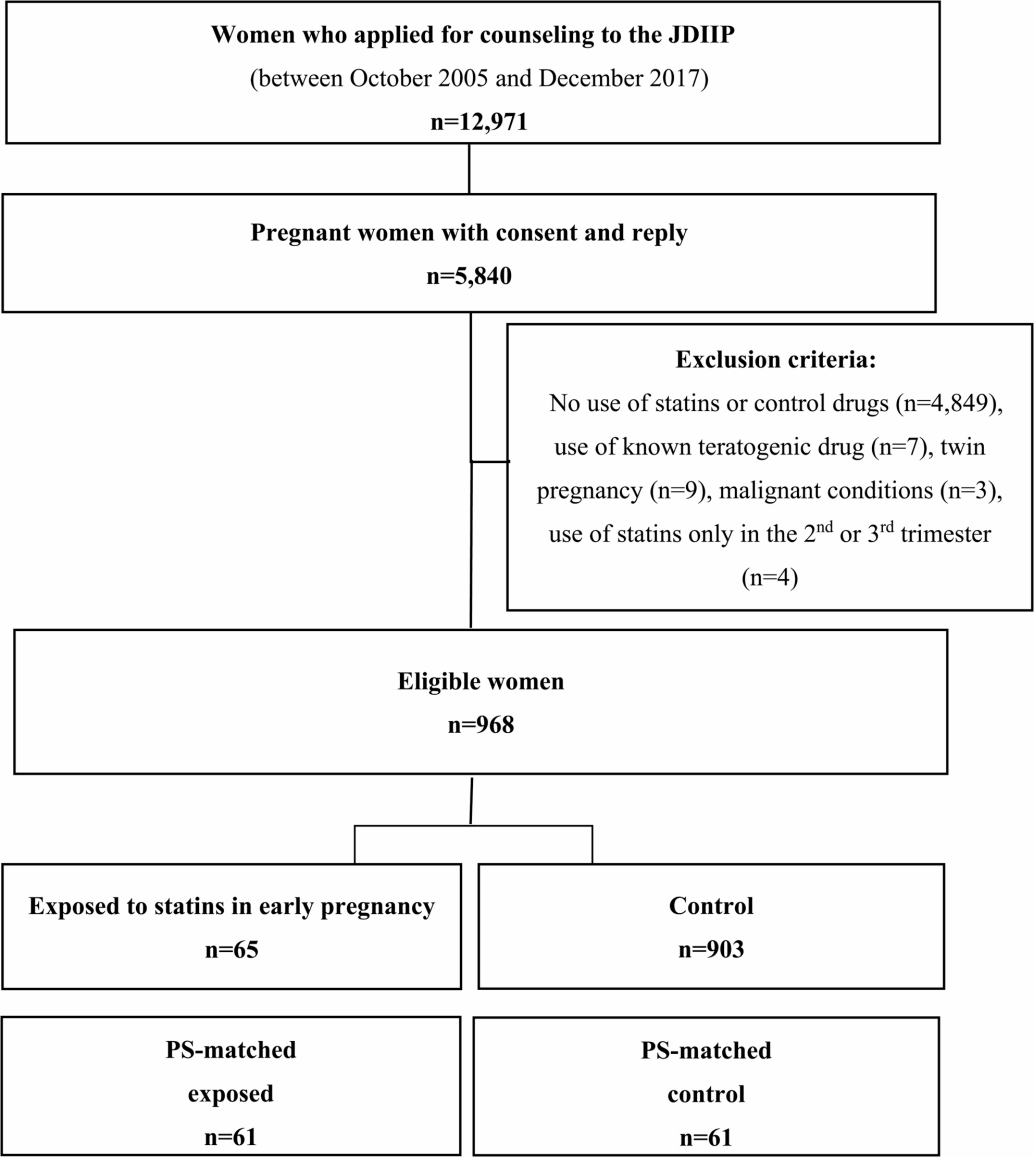
Study flow chart. JDIIP: Japan Drug Information Institute in Pregnancy, PS: propensity score

Among 5,840 pregnant women who received counseling at the JDIIP between 2005 and 2017, 968 who met the inclusion criteria and did not meet any exclusion criteria were included in the study. Of these, 65 women who used statins during early pregnancy were classified as the exposed group, whereas the remaining 903 comprised the control group (Fig. 1).

Table 1 presents the types of statins used during early pregnancy among the PS-matched exposed group, with atorvastatin, rosuvastatin, and pitavastatin being the most frequently used. In the crude analysis, women in the exposed group had a higher BMI and were more likely to have hypertension, diabetes mellitus and psychiatric disorders compared to those in the control group. However, after 1:1 PSM, while the imbalance in age and BMI was largely reduced, SMDs indicated residual imbalance for certain maternal comorbidities, such as hypertension, diabetes mellitus, and psychiatric disorders (Table 2). PSM improved the overlap in propensity score distributions between the exposed and control groups (Figure S1); specifically, 97% of the statin-exposed group and 95% of the control group fell within the region of common support, indicating substantial overlap between groups. Stabilized weight diagnostics from the IPW analysis showed no extreme values, and no trimming was required. However, residual imbalance remained for several key maternal comorbidities.

**Table 1:** Types of statins used during pregnancy

Type of Statins	*n*
Atorvastatin	25
Rosuvastatin	16
Pitavastatin	12
Pravastatin	9
Simvastatin	1
Fluvastatin	0

Type of statins that pregnant women were exposed to in early pregnancy among the propensity score-matched exposed group, including duplicates

**Table 2:** Maternal characteristics

	Crude			Propensity Score-Matched		
	Exposed to statins	Control	SMD	Exposed to statins	Control	SMD
	(*N* = 65)	(*N* = 903)		(*N* = 61)	(*N* = 61)	
Age (years)	34.0 (31.0, 38.0)	32.0 (29.0, 35.0)	0.622	34.0 (31.0, 38.0)	34.0 (30.0, 37.0)	0.183
<30	8 (12.3%)	287 (31,8%)	0.482	8 (13.1%)	8 (13.1%)	0.000
30–39	45 (69.2%)	583 (64.6%)	0.099	44 (72.1%)	47 (77.0%)	0.112
≥ 40	12 (18.5%)	33 (3.7%)	0.483	9 (14.8%)	6 (9.8%)	0.149
BMI (kg/m^2^)	21.6 (19.5, 24.6)	19.9 (18.6, 21.5)	0.636	21.5 (19.4, 22.9)	20.6 (19.7, 23.3)	0.091
< 18	7 (11.5%)	138 (15.3%)	0.134	7 (11.5%)	6 (9.8%)	0.053
18–25	43 (70.5%)	706 (78.2%)	0.270	43 (70.5%)	42 (68.9%)	0.035
> 25	15 (24.6%)	59 (6.5%)	0.476	11 (18.0%)	13 (21.3%)	0.081
Year of consultation						
2005–2009	13 (20.0%)	322 (36.8%)	0.354	13 (21.3%)	12 (19.7%)	0.040
2010–2014	29 (44.6%)	445 (49.3%)	0.093	29 (47.5%)	30 (49.2%)	0.031
2015–2017	23 (35.4%)	136 (15.1%)	0.479	19 (31.1%)	19 (31.1%)	0.000
Alcohol use during pregnancy	21 (32.3%)	426 (47.2%)	0.306	21 (34.4%)	20 (32.8%)	0.034
NA	0	1 (0.1%)	0.047	0	0	0.000
Smoking during pregnancy	6 (3.6%)	90 (2.1%)	0.025	6 (9.8%)	7 (11.5%)	0.053
NA	0	1 (0.1%)	0.047	0	0	0.000
Parous woman	36 (55.4%)	500 (55.4%)	0.000	33 (54.1%)	33 (54.1%)	0.000
Folic acid supplementation prior to pregnancy	10 (15.4%)	116 (12.9%)	0.073	10 (16.3%)	7 (11.5%)	0.141
Maternal comorbidities						
HTN, n (%)	6 (9.2%)	3 (0.3%)	0.426	4 (6.6%)	0	0.375
DM, n (%)	12 (18.5%)	1 (0.1%)	0.666	10 (16.4%)	0	0.626
PHY, n (%)	10 (15.4%)	20 (2.2%)	0.478	9(14.8%)	0	0.588
CVD, n (%)	2 (3.1%)	8 (0.9%)	0.158	2 (3.2%)	1 (1.6%)	0.106

Continuous variables are summarized as medians with interquartile ranges (IQRs), while categorical variables are presented as (percentages, %). The exposed statin group is defined as women who used statins during early pregnancy, while the control group comprises those who did not use statins at any point during pregnancy. Propensity score is estimated by logistic regression, with statin exposure as the dependent variable and age, BMI, registration year, alcohol use, and smoking as covariates. Alcohol use during pregnancy and smoking during pregnancy include both discontinued and ongoing use during pregnancy

*Abbreviation*: *PSM* propensity score-matched, *BMI* body mass index, *HTN* hypertension, *DM* diabetes mellitus, *PHY* psychiatric disorders, *CVD* cardiovascular disease, *SMD*  standardized mean difference

Regarding outcomes, after PSM, major congenital anomalies were reported in one case each in both the exposed (1.6%) and control (1.6%) groups, resulting in an odds ratio (OR) of 1.00 (95% confidence interval [CI]: 0.06–15.99) (Table 3). For preterm birth, the PSM odds ratio was 1.17 (95% CI: 0.39–3.47), whereas the IPW analysis yielded a risk ratio of 4.26 (95% CI: 2.02–8.99). Similarly, for low birth weight, the PSM odds ratio was 1.60 (95% CI: 0.52–4.89), while the IPW risk ratio was 3.32 (95% CI: 1.73–6.36) (Table 3; Table S1). A complete-case sensitivity analysis excluding participants with missing covariate information yielded consistent results (Table S2). In exploratory descriptive analyses within the matched cohort, no clear differences in outcomes were observed between hydrophilic and lipophilic statins (Table S4).

**Table 3:** Pregnancy outcomes in exposed and control group after propensity score matching

	Propensity Score-Matched			RD (%)
	Exposed to statins	Control	OR (95%CI)	
	(*N* = 61)	(*N* = 61)		
Live birth	52 (85.2%)	54 (88.5%)	OR 0.67 (0.18, 2.36)	-
Miscarriage	6 (9.8%)	5 (8.2%)	OR 1.25 (0.34, 4.65)	-
Abortion	2 (3.3%)	1 (1.6%)	OR 2.00 (0.18, 22.06)	-
Stillbirth	1 (1.6%)	1 (1.6%)	OR 1.00 (0.06, 15.99)	-
Preterm birth	8 (13.1%)	7 (11.5%)	OR 1.17 (0.39, 3.47)	+0.6
Low birth weight	11 (18.0%)	8 (13.1%)	OR 1.60 (0.52, 4.89)	+4.9
Anomaly, n (%)	4 (6.6%)	3 (4.9%)	OR 1.33 (0.30, 5.96)	+1.7
Major, n (%)	1 (1.6%)	1 (1.6%)	OR 1.00 (0.06, 15.99)	0.0
Minor, n (%)	3 (4.9%)	2 (3.3%)	OR 1.50 (0.25, 8.98)	+1.6

Propensity score is estimated by logistic regression, with statin exposure as the dependent variable and age, BMI, registration year, alcohol use, and smoking as covariates. Analyses of preterm birth and low birth weight were restricted to live births. RD (%), risk difference expressed as percentage points (Statin - Control), was calculated from observed proportions in the propensity score–matched sample

*Abbreviation*: *PSM* propensity score-matched, *OR* odds ratio, *RD* risk difference

Table 4 presents details of congenital anomalies observed in children within the statin-exposed group, along with information on the types of statins used, gestational age at which the mother discontinued statin use, gestational age at delivery, and types of concomitant medications. The types of statins used were atorvastatin and pitavastatin, and the congenital anomalies observed were distinct from each other.

**Table 4:** Details of congenital anomalies observed in the statin-exposed group

	Maternal age/diagnosis	Type of statins	Gestational weeks of last medicine use (weeks)	Pregnancy outcome	Gestational weeks of delivery (weeks)	Major or minor anomaly	Congenital malformation	Concomitant medication use during the first trimester
1	40 /FH	Atorvastatin	5	Stillbirth	28	Major	Complex cardiac malformation, fetal oedema	Ezetimibe
2	37 /SLE	Atorvastatin	5	Livebirth	38	Minor	Persistent foramen ovale	Risedronate, prednisolone, tacrolimus, teprenone, rabeprazole, aspirin, menatetrenone
3	33 / Unknown	Pitavastatin Atorvastatin was used 14 weeks before pregnancy	6	Livebirth	40	Minor	Undescended testicle	None
4	35 / Hyperlipidemia	Pitavastatin	8	Livebirth	37	Minor	Preauricular appendage	Sodium ferrous citrate, metformin, voglibose, magnesium oxide, lubiprostone, tokakujokito (kampo)

*Abbreviation*: *FH* familial hypercholesterolemia, *SLE* systemic lupus erythematosus

## Discussion

To the best of our knowledge, this is the first study in Japan to evaluate the impact of statin exposure during early pregnancy. Regarding congenital anomalies, our findings are consistent with those of previous studies [1, 2, 9, 11], supporting the existing evidence that statin exposure in early pregnancy is not associated with a substantially increased risk. In contrast, for perinatal outcomes, while no statistically significant differences were observed in the PS-matched analysis, weighted analysis using IPW showed higher risks of preterm birth and low birth weight in the statin-exposed group.

Regarding neonatal congenital anomalies, no statistically significant increased risk was observed with statin use during the early pregnancy; however, the wide confidence interval (0.06–15.99) reflects limited statistical power and substantial imprecision, and clinically important increases in risk cannot be excluded. In 2004, the FDA reported cases of birth defects in adverse event reports associated with statin exposure during pregnancy [20]. Subsequent studies, however, have not demonstrated a significant increase in the risk of congenital anomalies. The Motherisk program in North America reported 64 cases of statin exposure during early pregnancy [9], whereas 11 Teratology Information Services (TIS) in Europe reported 249 cases [21], both showing no significant difference in the incidence of birth defects between the statin-exposed and non-exposed groups. These studies share similarities with the present study in that accurate exposure information and perinatal outcomes were obtained through detailed consultation during early pregnancy, and a structured telephone interview or mailed questionnaire was completed by the woman or her physician.

Additionally, studies utilizing insurance databases have reported findings consistent with those from TIS, showing no increased risk of congenital anomalies associated with statin exposure during early pregnancy. For instance, a study using U.S. Medicaid claims data analyzed 1,152 cases of statin prescriptions during early pregnancy and found no significant difference in the risk of congenital anomalies compared to the non-exposure group [22]. Similarly, a study using a Taiwanese insurance database also reported no significant increase in risk [11]. A recent nationwide Korean study likewise reported no overall increase in congenital anomalies among statin-exposed pregnancies. Although the timing of exposure was not restricted specifically to the early pregnancy, many exposures likely occurred before pregnancy recognition, and no elevated risk was observed in these cases [23]. Consistent with these previous reports, we also found no increased risk of birth defects associated with early exposure to statins. The aforementioned studies primarily involved women with hypercholesterolemia who used statins until they became aware of their pregnancy and discontinued the medication thereafter. Considering the time required to transition from planning a pregnancy to actual conception, a prolonged period without lipid-lowering treatment could be disadvantageous for women with hypercholesterolemia who require continuous management. As highlighted by the U.S. FDA, statin use may not necessarily be contraindicated, particularly for women with hypercholesterolemia at high risk of cardiovascular disease.

In this study, early exposure to statins was associated with an increased incidence of preterm birth and low birth weight in the IPW analysis. Several studies have investigated the association between hyperlipidemia and adverse pregnancy outcomes. A prospective cohort study found that elevated maternal lipid levels including cholesterol and triglycerides were significantly associated with an increased risk of preterm delivery, and low lipid levels were also associated with preterm birth [24]. In contrast, women with FH do not appear to have a higher risk of preterm delivery or low birth weight, according to a large registry-based study from Norway [25]. Our study population included mothers with FH or hyperlipidemia who used statins in early pregnancy, although the cohort was not limited to FH cases. While our findings do not show a direct association between FH and adverse outcomes, maternal lipid abnormalities, including both low and high cholesterol levels, have been associated with preterm birth, potentially due to their impact on placental function [24]. Given that FH is characterized by persistent lipid abnormalities, these metabolic disturbances contribute to fetal growth restriction, which warrants further investigation [24].

Statistically significance was observed only in the IPW analysis, but both PSM and IPW showed a consistent direction of association, suggesting an increased risk in the statin-exposed group. The wide CIs in the PSM results suggest imprecision, likely due to the limited sample size and number of outcome events. In contrast, the larger point estimates observed in the IPW analysis may reflect the sensitivity of weighting methods to residual imbalance and limited clinical overlap between groups. When positivity is weak, extreme weights can disproportionately influence weighted estimates, potentially leading to inflated risk ratios. Therefore, these findings should be interpreted with caution. Notably, absolute risk differences were modest (1.6 and 4.9 percentage points for preterm birth and low birth weight, respectively).

Recently, pravastatin has been investigated for its potential to prevent preeclampsia and improve perinatal outcomes in women at high risk for hypertension disorder of pregnancy (HDP) [26–28]. Although HDP remains a major cause of maternal morbidity and mortality, and statins show promise in this context, their efficacy and safety remain under investigation, particularly regarding potential neurodevelopmental risks to the child. The use of statins beyond early pregnancy was not examined in this study, necessitating the need for further research to evaluate the safety of continuous statin use throughout pregnancy.

This study has several strengths. First, recall bias was minimized by using prospectively collected data from women who received counseling. Second, precise information on the timing of statin use allowed for accurate assessment of exposure during early pregnancy. Third, detailed data on confounders, such as smoking, alcohol consumption, and folic acid intake, were collected directly from participants. Fourth, maternal reports of child outcomes had a high response rate of 83.1% [12], supported by the study’s design, which fostered trust through counseling and information-sharing. Finally, leveraging a nationwide network enabled the collection of a substantial number of cases, even for a condition such as hypercholesterolemia, which is relatively uncommon among women of childbearing age.

This study also has limitations. First, self-reported data may include inaccuracies, particularly regarding pre-existing conditions and congenital anomalies. However, reports of congenital anomalies were largely based on pediatrician-documented records in the Maternal and Child Health Handbook during the one-month check-up, which has a high attendance rate in Japan. Additionally, follow-up phone calls were conducted to verify reported anomalies. Second, in our counseling and follow-up system, we collect maternal and neonatal information at 1 month after delivery. Therefore, we do not have data on maternal medication use after counseling during pregnancy or on neonatal congenital anomalies diagnosed after 1 month of age. Congenital anomalies were assessed at the 1-month follow-up; thus, our findings reflect anomalies detected early in life and may underestimate the total burden of congenital anomalies. Some anomalies, particularly certain cardiac, renal, or neurodevelopmental conditions, may be diagnosed after the neonatal period. Third, loss to follow-up may have included cases of non-live births, such as miscarriage or stillbirth, introducing potential selection bias. Although the number is likely small, non-random missingness cannot be entirely ruled out. In addition, because congenital anomaly analyses were restricted to live births, severe anomalies resulting in miscarriage, stillbirth, or elective termination may not have been fully captured, which could influence the observed associations (live-birth bias). If severe anomalies are more likely to result in pregnancy loss, this restriction could lead to underestimation of the true risk of congenital anomalies among statin-exposed pregnancies. Uncertainty in gestational age estimation based on LMP and/or EDD may have resulted in non-differential exposure misclassification, particularly clinical overlap among early pregnancy losses, biasing results toward the null. Fourth, the possibility of positivity violation should be considered, as the statin-exposed group included women with FH or hyperlipidemia, whereas such conditions were unlikely in the control group. In our consultation-based case database, patients with FH or severe hyperlipidemia who are not treated with statins are extremely rare, which also limits the feasibility of constructing a disease-matched unexposed control group. In a restricted exploratory subset limited to women with hypertension, diabetes mellitus, or cardiovascular disease, very few unexposed controls were available, precluding meaningful comparative estimation (Table S3). Under such conditions of limited clinical overlap, IPW estimates may be sensitive to model specification when positivity is weak and should therefore be interpreted with caution. Consequently, the observed associations should not be interpreted as evidence of a causal effect independent of the underlying disease. Finally, the data were derived from a consultation-based database in Japan, which includes women who actively sought counseling regarding medication use during pregnancy. Therefore, the study population may not fully represent the general population of pregnant women. Unmeasured confounding factors, such as education, income, and disease severity, were not assessed, which may have influenced the results. In addition, other factors not captured in our dataset—such as genetic predispositions, dietary habits, health-seeking behavior, and the severity of underlying dyslipidemia—may have affected both statin use and pregnancy outcomes, leading to potential residual confounding. Furthermore, because hypertension, diabetes mellitus, and other comorbidities are more common among women with hyperlipidemia treated with statins, residual imbalance in overall comorbidity burden may have influenced the observed associations, particularly for preterm birth and low birth weight.

## Conclusion

No increase was observed in congenital anomalies detected by 1 month among live births following early statin exposure, although the estimates were imprecise, as reflected by the wide confidence intervals. However, the results should be interpreted in the context of a consultation-based database in Japan and the possibility of residual confounding, including unmeasured socio-demographic factors. Nevertheless, these findings may provide reassurance regarding inadvertent early pregnancy exposure and support individualized shared decision-making for women of reproductive age who require statin therapy.

## Supplementary Information

Additional file 1: Table S1. Weighted analysis using the inverse probability weighting (IPW) method of pregnancy outcomes in the exposed and control groups after propensity score matching. Table S2. Complete-case propensity score–matched analysis corresponding to Table S1. Table S3. Descriptive outcomes in the restricted subset of women with hypertension, diabetes mellitus, or cardiovascular disease. Table S4. Exploratory descriptive outcomes according to statin lipophilicity in the propensity score–matched sample. Figure S1. Distribution of propensity scores in the statin-exposed and control groups before and after propensity score matching.

## Data Availability

The data were obtained from the Japan Drug Information Institute in Pregnancy (JDIIP) and are not publicly available due to privacy and ethical restrictions. Data may be available from the corresponding author upon reasonable request and with permission from the Japan Drug Information Institute in Pregnancy.

## Abbreviations

PSM: Propensity score matching
BMI: Body mass index
FH: Familial hypercholesterolemia
LDL: Low-density lipoprotein
JDIIP: Japan Drug Information Institute in Pregnancy
IQR: Interquartile ranges
IPQ: Inverse probability weighting
OR: Odds ratio
CI: Confidence interval
TIS: Teratology Information Services
HDP: Hypertension disorder of pregnancy

## References

[1] McGrogan A, Snowball J, Charlton RA. Statins during pregnancy: a cohort study using the General Practice Research Database to investigate pregnancy loss. Pharmacoepidemiol Drug Saf. 2017;26:843–52. 28176447 10.1002/pds.4176

[2] Lee MS, Hekimian A, Doctorian T, Duan L. Statin exposure during first trimester of pregnancy is associated with fetal ventricular septal defect. Int J Cardiol. 2018;269:111–3. 29996977 10.1016/j.ijcard.2018.07.002

[3] Graham DF, Raal FJ. Management of familial hypercholesterolemia in pregnancy. Curr Opin Lipidol. 2021;32:370–7. 34619689 10.1097/MOL.0000000000000790

[4] Mitsuda N, Eitoku M, Yamasaki K, J-p NA, Fujieda M, Maeda N, et al. Association between maternal cholesterol level during pregnancy and placental weight and birthweight ratio: data from the Japan Environment and Children’s Study. BMC Pregnancy Childbirth. 2023;23:484. 37391691 10.1186/s12884-023-05810-3 PMC10311780

[5] Grundy SM, Stone NJ, Bailey AL, Beam C, Birtcher KK, Blumenthal RS, AHA/ACC/AACVPR/AAPA/ et al. AHA/ACC/AACVPR/AAPA/ABC/ACPM/ADA/AGS/APhA/ASPC/NLA/PCNA guideline on the management of blood cholesterol: a report of the American College of Cardiology/American Heart Association Task Force on Clinical Practice Guidelines. J Am Coll Cardiol. 2018;73:e285–350 10.1016/j.jacc.2018.11.003 30423393

[6] US Food and Drug Administration. MEVACOR^®^ (lovastatin) Tablets, for oral use [Prescribing Information]. 2014. https://www.accessdata.fda.gov/drugsatfda_docs/label/2014/019643s088lbl.pdf.

[7] FDA requests removal of strongest warning. against using cholesterol-lowering statins during pregnancy; still advises most pregnant patients should stop taking statins; 2021. https://www.fda.gov/drugs/drug-safety-and-availability/fda-requests-removal-strongest-warning-against-using-cholesterol-lowering-statins-during-pregnancy.

[8] JA Society. The 2022 guidelines for the diagnosis and treatment of adult familial hypercholesterolemia (FH); 2022. https://square.umin.ac.jp/~jslpc/pdf/JAS_FH_GL2022.pdf. 10.5551/jat.CR005 PMC10164595 36682773

[9] Taguchi N, Rubin ET, Hosokawa A, Choi J, Ying AY, Moretti ME, et al. Prenatal exposure to HMG-CoA Reductase Inhibitors: effects on fetal and neonatal outcomes. Reprod Toxicol. 2008;26:175–7. 18640262 10.1016/j.reprotox.2008.06.009

[10] Makino H, Koezuka R, Tamanaha T, Ogura M, Matsuki K, Hosoda K, et al. Familial hypercholesterolemia and lipoprotein apheresis. J Atheroscler Thromb. 2019;26:679–87. 31231083 10.5551/jat.RV17033 PMC6711846

[11] Chang JC, Chen YJ, Chen IC, Lin WS, Chen YM, Lin CH. Perinatal outcomes after statin exposure during pregnancy. JAMA Netw Open. 2021;4:e2141321. 34967881 10.1001/jamanetworkopen.2021.41321 PMC8719244

[12] Yakuwa N, Murashima A, Miyazaki S. Investigation on the accumulation of background and pregnancy outcome information on cases consulted by the Japan Drug Information Institute in Pregnancy. Congenit Anom (Kyoto). 2024;64:6–16. 38072629 10.1111/cga.12547

[13] Murashima A, Yakuwa N, Koinuma S, Uno C, Takai C, Fujioka I, et al. The advances in dealing with the safety of medicated drugs in pregnancy. Glob Health Med. 2021;3:175–9. 34250294 10.35772/ghm.2020.01120 PMC8239373

[14] Goto M, Anzai T, Yamane R, Yakuwa N, Takahashi K, Murashima A. Pregnancy outcomes after first-trimester exposure to fluoroquinolones: findings based on an integrated database from two Japanese institutions. Congenit Anom (Kyoto). 2024;64:199–206. 38936845 10.1111/cga.12577

[15] Hishinuma K, Yamane R, Yokoo I, Arimoto T, Takahashi K, Goto M, et al. Pregnancy outcome after first trimester exposure to domperidone-An observational cohort study. J Obstet Gynaecol Res. 2021;47:1704–10. 33631840 10.1111/jog.14709 PMC8248151

[16] Hatakeyama S, Goto M, Yamamoto A, Ogura J, Watanabe N, Tsutsumi S, et al. The safety of pranlukast and montelukast during the first trimester of pregnancy: a prospective, two-centered cohort study in Japan. Congenit Anom (Kyoto). 2022;62:161–8. 35538631 10.1111/cga.12471

[17] Yamaguchi Y, Yamada T, Goto M, Kawasaki H, Wada T, Ikeda-Sakai Y, et al. Analysis of triptan use during pregnancy in Japan: A case series. Congenit Anom (Kyoto). 2022;62:78–81. 34981573 10.1111/cga.12456

[18] Yakuwa N, Takahashi K, Anzai T, Ito N, Goto M, Koinuma S, et al. Pregnancy outcomes with exposure to second-generation antipsychotics during the first trimester. J Clin Psychiatry. 2022;83:21m14081. 35687862 10.4088/JCP.21m14081

[19] U.o.U. Instruction for the Registration of Congenital Anomalies. EUROCAT Central Registry, EUROCAT Guide 1.4 and Reference Documents (Last update version 07/10/2021), 2021.

[20] Edison RJ, Muenke M. Central nervous system and limb anomalies in case reports of first-trimester statin exposure. N Engl J Med. 2004;350:1579–82. 15071140 10.1056/NEJM200404083501524

[21] Winterfeld U, Allignol A, Panchaud A, Rothuizen LE, Merlob P, Cuppers-Maarschalkerweerd B, et al. Pregnancy outcome following maternal exposure to statins: a multicentre prospective study. BJOG. 2013;120:463–71. 23194157 10.1111/1471-0528.12066

[22] Bateman BT, Hernandez-Diaz S, Fischer MA, Seely EW, Ecker JL, Franklin JM, et al. Statins and congenital malformations: cohort study. BMJ. 2015;350:h1035. 25784688 10.1136/bmj.h1035 PMC4362473

[23] Kay HY, Jang HY, Kim IW, Oh JM. Pregnancy and neonatal outcomes after fetal exposure to statins among women with dyslipidemia: a nationwide cohort. Eur J Pediatr. 2025;184:340. 40366447 10.1007/s00431-025-06119-3 PMC12078441

[24] Mudd LM, Holzman CB, Catov JM, Senagore PK, Evans RW. Maternal lipids at mid-pregnancy and the risk of preterm delivery. Acta Obstet Gynecol Scand. 2012;91:726–35. 22404756 10.1111/j.1600-0412.2012.01391.x PMC4563824

[25] Toleikyte I, Retterstøl K, Leren TP, Iversen PO. Pregnancy outcomes in familial hypercholesterolemia: a registry-based study. Circulation. 2011;124:1606–14. h. 21911783 10.1161/CIRCULATIONAHA.110.990929

[26] Ahmed A, Williams DJ, Cheed V, Middleton LJ, Ahmad S, Wang K, et al. Pravastatin for early-onset pre-eclampsia: a randomised, blinded, placebo-controlled trial. BJOG. 2020;127:478–88. 31715077 10.1111/1471-0528.16013 PMC7063986

[27] Costantine MM, West H, Wisner KL, Caritis S, Clark S, Venkataramanan R, et al. A randomized pilot clinical trial of pravastatin versus placebo in pregnant patients at high risk of preeclampsia. Am J Obstet Gynecol. 2021;225:e6661–15. 10.1016/j.ajog.2021.05.018 PMC8611118 34033812

[28] Kumasawa K, Iriyama T, Nagamatsu T, Osuga Y, Fujii T. Pravastatin for preeclampsia: from animal to human. J Obstet Gynaecol Res. 2020;46:1255–62. 32485787 10.1111/jog.14295

