# Supplementary material for: Pregnancy and neonatal outcomes following statin exposure in early pregnancy: a nationwide consultation-based cohort study in Japan
Source: BMC Pregnancy Childbirth. 2026 Mar 14;26:449. doi: 10.1186/s12884-026-08932-6 (PMC13101253; doi:10.1186/s12884-026-08932-6)
Supplement: Supplementary file 1 — Additional file 1: Table S1. Weighted analysis using the inverse probability weighting (IPW) method of pregnancy outcomes in the exposed and control groups after propensity score matching. Table S2. Complete-case propensity score–matched analysis corresponding to Table S1. Table S3. Descriptive outcomes in the restricted subset of women with hypertension, diabetes mellitus, or cardiovascular disease. Table S4. Exploratory descriptive outcomes according to statin lipophilicity in the propensity score–matched sample. Figure S1. Distribution of propensity scores in the statin-exposed and control groups before and after propensity score matching. [file 12884_2026_8932_MOESM1_ESM.zip › Supplementary_Materials_R1_clean.docx]

**Additional File 1**

*****

**Pregnancy and Neonatal Outcomes Following Statin Exposure in Early Pregnancy: A Nationwide Consultation-Based Cohort Study in Japan**

**Izumi Fujioka, Mikako Goto, Tatsuhiko Anzai, Kunihiko Takahashi,**

**Sachi Koinuma, Atsuko Murashima**

*****

**Contents**

| **Table S1. Weighted analysis using the inverse probability weighting (IPW) method of pregnancy outcomes in the exposed and control groups after propensity score matching** | **2** |
| --- | --- |
| **Table S2. Complete-case propensity score–matched analysis corresponding to Table S1** | **3** |
| **Table S3. Descriptive outcomes in the restricted subset of women with hypertension, diabetes mellitus, or cardiovascular disease** | **4** |
| **Table S4. Exploratory descriptive outcomes according to statin lipophilicity in the propensity score–matched sample** | **4** |
| **Figure S1. Distribution of propensity scores in the statin-exposed and control groups before and after propensity score matching** | **5** |
|  |  |

**Table S1. Weighted analysis using the inverse probability weighting (IPW) method of pregnancy outcomes in the statin exposed and control groups**

| **Outcomes** |  | **Risk ratio (95% CI)** | **P-value** |
| --- | --- | --- | --- |
|  |  |  |  |
| **Live birth** |  | 0.99 (0.92, 1.08) | 0.905 |
| **Miscarriage** |  | 1.02 (0.37, 2.86) | 0.965 |
| **Abortion** |  | 0.73 (0.14, 3.77) | 0.707 |
| **Stillbirth** |  | 4.03 (0.41, 39.33) | 0.231 |
| **Preterm birth** |  | 4.26 (2.02, 8.99) | <0.001 |
| **Low birth weight** |  | 3.32 (1.73, 6.36) | <0.001 |
|  |  |  |  |
| **Anomaly,** |  | 1.98 (0.71, 5.54) | 0.192 |
| Major |  | 0.99 (0.97 , 1.01) | 0.213 |
| Minor |  | 4.61 (1.36, 15.61) | 0.014 |

Weighted analysis is performed using the inverse probability weighting (IPW) based on the total included sample (N = 968). Risk ratio and 95% confidence intervals are estimated using robust variance estimation. **Covariates adjusted for**: *age, year of consultation, alcohol use, smoking, parity, body mass index (BMI), diabetes, hypertension, psychiatric disorders, cardiac diseases, and folic acid supplementation prior to pregnancy.* Missing values were handled using a missing-indicator approach. The sum of the weights was 897.7 in the control group and 62.1 in the statin-exposed group.

**Table S2. Complete-case propensity score–matched analysis corresponding to Table S1**

| **Outcomes** |  | **Risk ratio (95% CI)** | **P-value** |
| --- | --- | --- | --- |
|  |  |  |  |
| **Live birth** |  | 0.99 (0.91, 1.09) | 0.910 |
| **Miscarriage** |  | 1.03 (0.35, 3.02) | 0.962 |
| **Abortion** |  | 0.76 (0.13, 4.39) | 0.757 |
| **Stillbirth** |  | 3.84 (0.34, 42.91) | 0.274 |
| **Preterm birth** |  | 4.10 (1.74, 9.64) | 0.001 |
| **Low birth weight** |  | 3.23 (1.57, 6.65) | 0.001 |
|  |  |  |  |
| **Anomaly,** |  | 1.98 (0.58, 6.79) | 0.275 |
| Major |  | 0.99 (0.97, 1.01) | 0.210 |
| Minor |  | 4.64 (1.10, 19.51) | 0.036 |

Complete-case analysis based on participants with complete covariate information (N = 966), excluding two control participants with missing information on alcohol consumption or smoking. Matching procedure, weighting method, and covariates adjusted were identical to those described in Table S1.**Table S3. Descriptive outcomes in the restricted subset of women with hypertension, diabetes mellitus, or cardiovascular disease**

| **Outcome** | **Statin (n=15)** | **Control (n=12)** |
| --- | --- | --- |
| Live birth | 11 (73.3%) | 11 (91.7%) |
| Major congenital anomaly  (1-month) | 0 (0.0%) | 0 (0.0%) |
| Preterm birth | 1 (6.7%) | 0 (0.0%) |
| Low birth weight | 0 (0.0%) | 1 (8.3%) |

This exploratory subset analysis was severely underpowered due to the very small number of clinically comparable unexposed controls. No effect estimates were calculated because of sparse outcome events. These results are presented for descriptive purposes only.

**Table S4. Exploratory descriptive outcomes according to statin lipophilicity in the propensity score–matched sample**

| **Outcome** | **Hydrophilic (n=25)** | **Lipophilic (n=36)** |
| --- | --- | --- |
| Live birth | 21 (84.0%) | 31 (86.1%) |
| Major congenital anomaly  (1-month) | 0 (0.0%) | 1 (2.8%) |
| Preterm birth | 3 (12.0%) | 5 (13.9%) |
| Low birth weight | 3 (12.0%) | 8 (8.3%) |

Hydrophilic statins included pravastatin and rosuvastatin; lipophilic statins included atorvastatin, pitavastatin, simvastatin, and fluvastatin. Outcomes are descriptively presented within the propensity score–matched cohort without formal statistical comparison and should be considered exploratory.

**Figure S1. Distribution of propensity scores in the statin-exposed and control groups before and after propensity score matching.**

**Before PSM (Propensity score distributions before matching)**


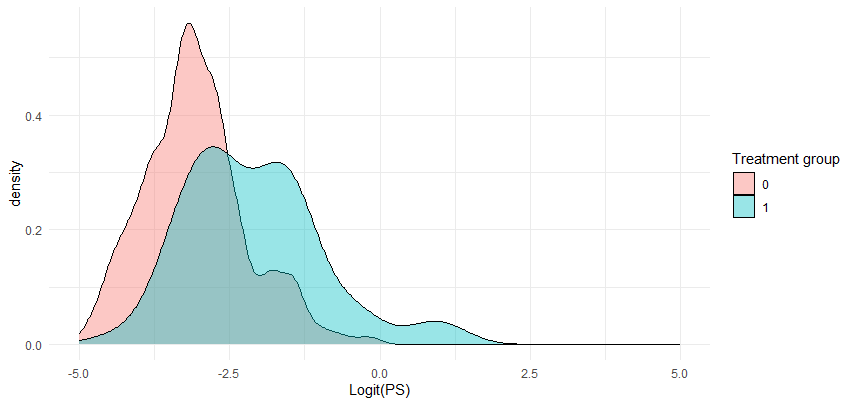


|  | min | 25% tile | 75% tile | Max |
| --- | --- | --- | --- | --- |
| Control | 0.0000005 | 0.0243206 | 0.0617480 | 0.4729890 |
| Statin | 0.01163 | 0.04826 | 0.19374 | 0.76380 |

**After PSM (Propensity score distributions after matching)**


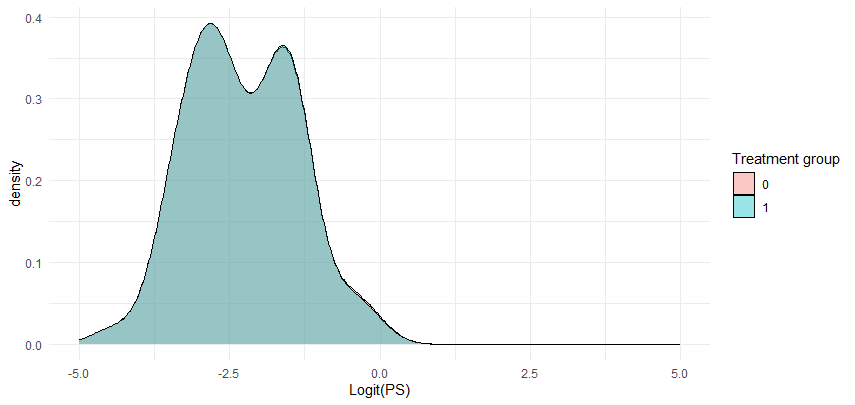


|  | min | 25% tile | 75% tile | Max |
| --- | --- | --- | --- | --- |
| Control | 0.01163 | 0.04826 | 0.18779 | 0.46968 |
| Statin | 0.01163 | 0.04826 | 0.18779 | 0.46968 |

Group classification: 0 = Control group, 1 = Statin-exposed group. PSM, propensity score matching

Values represent the minimum, 25th percentile, 75th percentile, and maximum of the estimated propensity scores in each group.
